# mGluR-dependent plasticity in rodent models of Alzheimer’s disease

**DOI:** 10.3389/fnsyn.2023.1123294

**Published:** 2023-03-02

**Authors:** Gonzalo Valdivia, Alvaro O. Ardiles, Abimbola Idowu, Claudia Salazar, Hey-Kyoung Lee, Michela Gallagher, Adrian G. Palacios, Alfredo Kirkwood

**Affiliations:** ^1^Mind/Brain Institute and Department of Neurosciences, Johns Hopkins University, Baltimore, MD, United States; ^2^Centro Interdisciplinario de Neurociencia de Valparaíso, Facultad de Ciencias, Universidad de Valparaíso, Valparaíso, Chile; ^3^Department of Psychological and Brain Sciences, Johns Hopkins University, Baltimore, MD, United States

**Keywords:** LTP (long term potentiation), LTD (long term pepression), mGluR5, Alzheimer’s disease, aging

## Abstract

Long-term potentiation (LTP) and depression (LTD) are currently the most comprehensive models of synaptic plasticity models to subserve learning and memory. In the CA1 region of the hippocampus LTP and LTD can be induced by the activation of either NMDA receptors or mGluR5 metabotropic glutamate receptors. Alterations in either form of synaptic plasticity, NMDAR-dependent or mGluR-dependent, are attractive candidates to contribute to learning deficits in conditions like Alzheimer’s disease (AD) and aging. Research, however, has focused predominantly on NMDAR-dependent forms of LTP and LTD. Here we studied age-associated changes in mGluR-dependent LTP and LTD in the APP/PS1 mouse model of AD and in *Octodon degu*, a rodent model of aging that exhibits features of AD. At 2 months of age, APP/PS1 mouse exhibited robust mGluR-dependent LTP and LTD that was completely lost by the 8th month of age. The expression of mGluR protein in the hippocampus of APP/PS1 mice was not affected, consistent with previous findings indicating the uncoupling of the plasticity cascade from mGluR5 activation. In *O. degu*, the average mGluR-LTD magnitude is reduced by half by the 3*^rd^* year of age. In aged *O. degu* individuals, the reduced mGluR-LTD correlated with reduced performance in a radial arm maze task. Altogether these findings support the idea that the preservation of mGluR-dependent synaptic plasticity is essential for the preservation of learning capacity during aging.

## Introduction

Alzheimer’s disease (AD) is a progressive neurodegenerative disorder that initially manifests as a severe impairment in memory formation. Consequently, much research interest focuses on alterations in synaptic mechanisms subserving the remodeling of brain circuitry during learning and memory formation. A wealth of studies on animal models, particularly mouse lines carrying genes of familial AD (FAD) have revealed clear impairments in the induction of Hebbian synaptic plasticity, specifically NMDAR-dependent forms of long-term potentiation (LTP) and long-term depression (LTD) (Selkoe, 2008; Ondrejcak et al., 2010; Mango et al., 2019; Zhang et al., 2022). Indeed, it has long been a widespread idea that disrupted synaptic plasticity contributes to initiating the AD pathological cascade (Selkoe, 2002). It must be noted, however, that reduced NMDAR-dependent synaptic plasticity is not unique to the AD condition, but a common consequence of aging. For example, both NMDAR-LTP and NMDAR-LTD are both reduced in aged Long-Evans rats (Lee et al., 2005; Boric et al., 2008), a non-pathological model of aging that does not exhibit AD signatures (Gallagher et al., 2003).

Besides NMDAR-dependent plasticity, LTD and LTP can also be induced by mechanisms requiring the activation of the metabotropic glutamate receptor 5 (Palmer et al., 1997; Huber et al., 2000; Wang et al., 2016). These two distinct systems of bidirectional plasticity are independent, complementary of each other, and can co-exist in the same synapse. Importantly, in models of non-pathological aging model what distinguishes learning-impaired individuals from non-impaired ones is their capacity to upregulate mGluR-dependent plasticity to compensate for the age-related reduction in NMDAR-dependent plasticity (Lee et al., 2005; Boric et al., 2008; Lu et al., 2019).

In contrast to NMDAR-dependent plasticity, the status of mGluR-dependent plasticity in models of AD remains largely unexplored. This is despite the fact that Aβ amyloid peptide oligomers, a leading candidate player in the pathological cascade (Li and Selkoe, 2020), stimulate internalization of AMPARs in a fashion comparable to mGluR-LTD (Kamenetz et al., 2003; Hsieh et al., 2006; Menard and Quirion, 2012a,b). Motivated by this paucity of information we examined the induction of mGluR-dependent synaptic plasticity in rodent models: the well-characterized APP/PS1 mouse (APPswe;PS1ΔE9) carrying two FAD mutations and *Octodon Degu*. *O degu* is a Chilean rodent model in which by the 3rd year of age some individuals spontaneously began expressing several signatures of AD including elevated levels of Aβ amyloid peptide oligomers associated with reduced memory performance and reduced NMDAR-LTP (Ardiles et al., 2012). We found that mGluR-dependent plasticity was absent in 8-month-old APP/PS1 mice, while it was reduced and associated with memory impairment in *O. degu* older than 30 months of age.

## Materials and methods

### Animals

Young (2-month-old) and adult (8-month-old) APPswe;PS1ΔE9 Tg and their wild-type (WT) littermate mice (129/C57BL/6 mixed background) of both sexes were used. APPswe;PS1ΔE9 Tg mice have accelerated amyloid pathologies and have a substantial number of plaque deposits by 6 months of age (Jankowsky et al., 2004; Savonenko et al., 2005). Therefore, these mice were used at both pre-amyloidogenic and post-amyloidogenic ages. All procedures performed with these animals were approved by the Institutional Animal Care and Use Committees of Johns Hopkins University.

Degus came from an inbreeding colony in the animal facility of the University de Valparaíso. Animals were kept at a controlled temperature (22 ± 1°C), in light/dark cycles (12:12) with water and food *ad libitum*. Animals of both sexes were used in age groups of 7–36 months (young), and 49–96 months (old). All procedures performed with these animals were approved according to the institutional program of animal care and use of the University de Valparaíso, Chile.

### Synaptic responses

Hippocampal slices (400 μm) were prepared as described previously (Boric et al., 2008; Ardiles et al., 2012; Megill et al., 2015). Before isolating the brain, the subjects were transcardially perfused, under isoflurane anesthesia with ice-cold dissection buffer containing: 212.7 mM sucrose, 2.6 mM KCl, 1.23 mM NaH_2_PO_4_, 26 mM NaHCO_3_, 10 mM dextrose, 3 mM MgCl_2_, and 1 mM CaCl_2_ bubbled with a mixture of 5%CO_2_ and 95%O_2_ and recovered at room temperature in Artificial Spinal Cerebral Fluid (ACSF) that replaced the sucrose by 124 mM NaCl. Synaptic responses were evoked with 0.2 ms pulses (10–80 μA) delivered through concentric bipolar theta glass micropipettes filled with ACSF, recorded extracellularly in CA1, and quantified as the initial slope of the field potential. Baseline responses were recorded at 0.033 Hz using a stimulation intensity that evoked a half-maximal response, defined as the maximal response without a population spike (pop-spike). Slices were discarded when the pop-spike appeared in the initial rising phase (an indication of hyperexcitability), when paired-pulse facilitation at a 50 ms interval was less than ∼10% (i.e., response 2/response 1 < 1.1), or when the baseline was not stable (∼5% drift). High-frequency tetanus consisted of four 200 Hz epochs (0.5 s) delivered at 0.2 Hz (in the presence of an NMDA receptor antagonist (100 μM D, L-APV). Under our experimental conditions, this protocol induces robust LTP in Long–Evans rats in both CA1 (Boric et al., 2008) and CA3 synapses (Yang et al., 2013; Wang et al., 2016). mGluR-LTD was induced using a paired-pulse 1 Hz (50 ms ISI) 900 pulse stimulation train in the presence of 100 μM APV. LTP and LTD magnitudes were calculated as the average (normalized to baseline) of the responses recorded 50–60 min after conditioning stimulation, corresponding to the early phase of LTP (to distinguish it from late LTP). Drugs included 2-Methyl-6-(phenylethynyl) pyridine (MPEP), LY-367385, (2R)-amino-5-phosphonovaleric acid (APV) from Tocris Bioscience; all other chemicals were from Sigma or Fisher Scientific.

### Western blot analysis

About 6–9 month-old of both genotypes were used (APPswe/PS1ΔE9 and Wild Type). Following decapitation under isoflurane anesthesia, the brains were quickly removed and placed in dissection buffer at 4°C to remove the hippocampus. Each hemisphere was placed in an Eppendorf tube on dry ice. Five hundred microliter of 1X lysis buffer was added immediately after (lysis buffer: 20 mM Phosphate buffer pH 7.4, 150 mM NaCl, 10 mM EDTA, 10 mM EGTA, 10 mM NaPPi, 50 mM NaF, 1 mM NaCO_3_) with 1% Triton X-100, protease inhibitor (Aprotinin 10 U/mL) and phosphatase inhibitor (1 μM okadaic acid). The samples were sonicated (Sonifier 450, Branson Ultrasonics) with 10 pulses in a cold room (4°C). SDS was then added to a concentration of 0.2%, and the samples were rotated (Rotisserie, Labquake) for 30 min to ensure complete lysis. The homogenate was centrifuged at 100 *g* (model 5415R centrifuge, Eppendorf) for 10 min. The supernatant was collected, and the protein concentration was calculated through the BCA assay (BCA Kit 23250, Pierce) using a spectrophotometer (Biophotometer model, Eppendorf). To 400 ml of sample, 200 ml of 3X GSB was added, and the protein concentration was normalized to 1.33 μg using 1X GSB (2% SDS (w/v), 10% glycerol (v/v), 5% β-Meracaptoethanol (v/v) in 0.125 M Tris, pH 6.8).

For gel electrophoresis, 16 μg of protein per sample was loaded in 6% polyacrylamide gel using 1X Tris-Glycine running buffer (25 mM Tris, 192 mM glycine, 0.1% SDS, pH 8.8). Protein separation was performed at 100 V for 1 h and 30 min.

Gels were transferred to PVDF membranes (ImmunBlot, Bio-Rad) at 26 V overnight using 1× Tris-Glycine blotting buffer with 20% methanol. The membranes were then fixed in destain (25% methanol, 10% acetic acid) for 15 min and blocked with blocking buffer (1% BSA, 0.1% Tween-20 in PBS, pH 7.4) for 1 hfff at room temperature.

Then, membranes were incubated with the primary antibodies anti-β-tubulin and anti-mGluR5 for 1 h at room temperature. After five washes with PBS-T, membranes were incubated with secondary antibodies conjugated to the fluorescent probe Cy3 and Cy5, which allowed the detection of anti-β-tubulin and anti-mGluR5, respectively, for 1 h at room temperature. Finally, membranes were washed with PBS-T and stored in the dark until detection. Blots were detected and scanned by a fluorescent scanner (Model 9419, Typhoon) and analyzed using the Image-J program (NIH).

### Behavioral analysis

For evaluating hippocampal-dependent spatial working memory in degus a Radial Arm Maze task was performed. The maze consists of an octagon with access to 8 arms (60 cm long), made of transparent methacrylate, and located 1 m above the ground. The experimental room had several external 3D visual keys that help the spatial orientation of the animal.

The behavioral protocol was based on a previous study (Chappell et al., 1998); this consisted of 4 stages*:* (i) *habituation:* the animal visited the maze for 10 min a day for 4 days. (ii) *Training phase*: animals seek a reward at the end of the arms once a day for 18 days. Each trial is finished when an animal visits the 8 arms or after 10 min of testing. (iii) *Test*: three trials were performed per day, for 9 days. The test consists of 2 phases, information phase (IP), where 4 arms are closed, and the animal visits open arms. This phase ends when the animal visits the 4 open arms or after 10 min. After 60 s the animal performs a memory phase (MP), where the 8 arms are open and the reward is present in the 4 arms that were kept closed during IP. In this last phase, retroactive memory (RAM) errors were evaluated, which are considered each time the animal enters an arm that had already visited during IP. The trial was finished when the animal visits the 4 arms that had the reward or after 10 min. The animals were positioned in the maze center for each trial in all the phases. The percentage of retroactive memory (RAM) error is determined for each trial, where an error of 0% corresponds to visiting the 4 arms not visited during IP and an error of 100% corresponds to visiting the 4 arms already visited in IP. Finally, for each animal, the RAM error was calculated as the mean of the 27 trials performed for MP.

### Statistics

Normality was determined by the Shapiro–Wilk test using Prism (GraphPad Software, San Diego, CA, USA). Mann–Whitney, *t*-Test, Kruskal–Wallis test, or ANOVA test were performed according to normality and the number of comparisons using Prism. Multiple comparisons were followed by the *post-hoc* Holm–Sidak test. Data are presented as averages ± SEM.

## Results

We examined the status of mGluR-dependent synaptic plasticity in two rodent models of aging, the APP/PS1 transgenic mouse model of AD (Jankowsky et al., 2004) and the Chilean rodent *Octodon degus*, which exhibits some traits associated with AD (Ardiles et al., 2012). To that end, we employed extracellular field potential recording methods (see methods for details) to test the induction of mGluR-LTD and mGluR-LTP in the Schaffer collateral to CA1 pathway in acute 400 μm slices.

**FIGURE 1:**
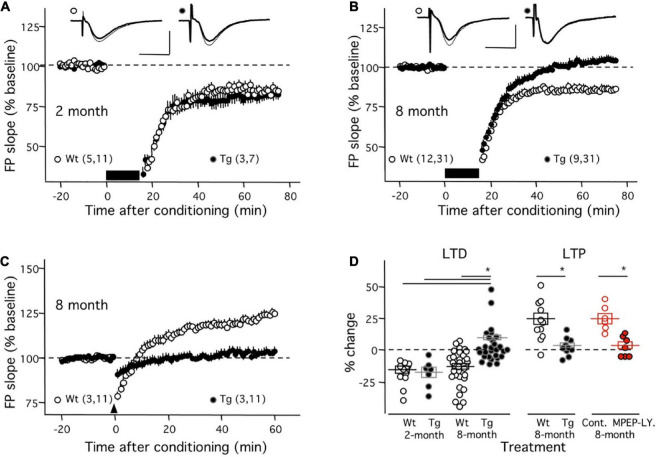
Absence on mGluR-dependent synaptic plasticity in middle-aged APP/PS1 transgenic mice. The graphs in panels **(A–C)** compare the time course of mGluR-LTD and mGluR-LTP induced in APP/PS1 transgenic mice (Tg: black circles) and age-matched control wild-type littermates (Wt: white circles). **(A)** In slices from young mice (2 months old) there is no difference in mGluR-LTD induced with ppLFS (black horizontal bar) between Wt and Tg mice. Example experiments are depicted on the top (Wt: left; Tg: right). The superimposed traces are averages of 4 consecutive responses recorded right before (thin line) and 60 min after conditioning (thick line). **(B)** At 8 months of age, ppLFS induces robust mGluR-LTD in slices from WT mice, but not from Tg mice. Example experiments are shown at the top with conventions as in panel **(A)**. **(C)** At 8 months of age the 200 Hz tetanus elicits robust mGluR-LTP in Wt but not Tg mice. **(D)** Summary results showing the magnitudes of mGluR-LTD and mGluR-LTP (50–60 min after conditioning) for all individual experiments in panels **(A–C)** (black symbols; Wt: open symbols; Tg: filled symbols) and also the blockade of mGluR-LTP by mGluR5 antagonists (red symbols) in slices from 8-month old WT mice (control: open circles; 10 mM MPEP and 100 mM LY367385: filled circles). Boxes in panel **(D)** and data points in panels **(A–C)** depict average ± SEM. Asterisks denote significance (*p* < 0.005). The number of mice and slices is indicated in parentheses). Calibration bars in panels **(A,B)**: 10 ms, 1 mV.

In the first set of experiments, we evaluated the induction mGluR-LTD (with ppLFS in the presence of 100 μM APV in the ACSF) in the APP/PS1 transgenic mouse (Tg). As shown in Figure 1A, in young (2-month-old) mice ppLFS induced robust mGluR-LTD in Tg individuals (81.64 ± 4.07% of initial baseline at 50–60 min after conditioning; *n* = 3 mice, 7 slices), which was comparable to the magnitude of mGluR-LTD induced in age-matched control wild type (WT) littermates (81.94 ± 2.94%; *n* = 5 mice, 11 slices; *p* = 0.9523, Mann–Whitney test). In contrast, as shown in Figure 1B, in slices from 8-month mice, when cognitive deficits and alterations in NMDAR-dependent plasticity are well developed in the Tg line (Megill et al., 2015), the induction of mGluR-LTD was virtually absent in Tg mice (104.2 ± 2.31%; *n* = 9, 31) and still robust in age-matched control WT mice (85.94 ± 2.48, *n* = 12, 31). A Kruskal–Wallis test (KW = 37.02; p < 0.0001) followed by a Dunn’s multiple comparison test confirmed the difference in the magnitude of mGluR-LTD between 8-month Tg mice and all the other groups (Figure 1D). Moreover, the effect size of the differences was very large (Cohen’s d value = 1.37). These results indicate a virtual complete age-dependent loss of mGluR-LTD in CA1 synapses of the APP/PS1 transgenic mouse model of AD.

In a subsequent set of studies, we evaluated the induction of mGluR-LTP (with repeated 200 Hz tetanus in the presence of 100 μM APV in the ACSF) in the APP/PS1 model at 8 months of age. Consistent with previous observations done in slices of younger individuals (Wang et al., 2016), the conditioning tetanus induced robust LTP in the slices from WT individuals (122.8 ± 4.67%, *n* = 11 slices from 3 mice). In contrast, Figure 1C shows that little changes were elicited in the slices of age-matched Tg mice (102.8 ± 1.98%, *n* = 11 slices from 3 mice). A t-test firmed the significance of these differences (*p* = 0.012). Finally, we confirmed that, like in slices from younger individuals (Wang et al., 2016), in slices from 8-month WT mice, this form of LTP requires mGluR5 function, as it was fully blocked by a combination of mGluR5 antagonists (Figure 1D; Control: 125.2 ± 4% change, *n* = 6 slices; in 110 μM MPEP and 100 μM LY341495 02.9 ± 3.0%, *n* = 7 slices; Mann–Whitney test: *p* = 0.0023). The effect size of these differences was large (Cohen’s d value = 1.19). Altogether the results indicate that at 8 months of age, the induction either of mGluR-LTP or mGluR-LTD is virtually absent in CA1 synapses from APP/PS1 transgenic mice.

**FIGURE 2:**
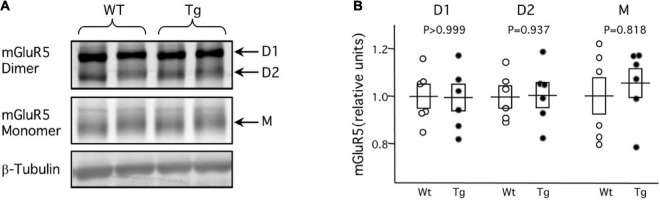
Normal expression of mGluR protein in middle-aged APP/PS1 transgenic mice. Western blot analysis of mGluR5 content in hippocampal tissue obtained from 8-month APP/PS1 transgenic mice (Tg) and age-matched control wild-type littermates. **(A)** Example blot showing lanes loaded with tissue from Wt (left two lanes) and Tg (right lanes) mice. D1 and D2 correspond to dimers with molecular weights of 290 and 260 KDa, respectively; M corresponds to the 130 KDa monomers. **(B)** Summary of the quantification of D1, D2, and M in 6 Wt mice (white circles) and 6 Tg mice (black circles). For each group (D1, D2, M) individual data points are normalized to the average value of the corresponding Wt. Boxes depict average ± SEM.

The virtual complete loss of mGluR-LTP and mGluR-LTD at 8-month in the APP/PS1 Tg mice suggests alterations early in the plasticity pathway, at stages that are common to LTP and LTD. In that context, it was of interest to evaluate possible changes in the expression mGluR5 protein in the APP/PS1 Tg mice. To that end, we determined with Western blots the mGluR5 protein content in the hippocampus of six 8-month WT and six Tg mice. The results, shown in Figure 2, revealed no differences between WT and Tg mice in the content of either the monomeric (WT: 1.00 ± 0.04, Tg: 1.00 ± 0.04; *p* > 0.999, Mann–Whitney test) or the D1 (WT: 1.00 ± 0.08, Tg: 0.99 ± 0.05; *p* = 0.937, Mann–Whitney test) and D2 (WT: 1.01 ± 0.05, Tg: 1.06 ± 0.06; *p* = 0.818, Mann–Whitney test) dimeric forms of the mGluR5. This similar content of mGluR5 in WT and Tg mice suggests an impairment downstream in the pathway.

**FIGURE 3:**
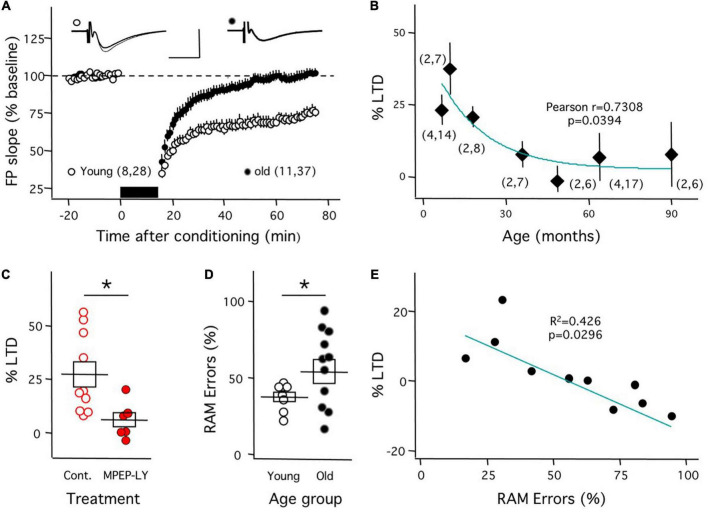
Reduced mGluR-LTD correlates with diminished performance in the radial arm maze in aged *O. degu*. Decline in mGluR-LTD in *O. degus.* **(A)** ppLFS induces robust mGluR-LTD in slices mGluR-LTD in slices from young (7–18 months-old: white circles) individuals, but from aged (49–94 months-old: black circles) individuals. Example experiments are depicted on the top (Young: left; Old: right). The superimposed traces are averages of 4 consecutive responses recorded right before (thin line) and 60 minutes after conditioning (thick line). **(B)** The average magnitude of mGluR-LTD (measured 60 min after conditioning) declines with age. The curve was drawn for illustrative purposes. **(C)** Blockade of mGluR-LTD by mGluR5 antagonists in slices from young *O. degus* (control: open circles; 10mM MPEP&100mM LY367385: filled circles). **(D)** Aged (49-94 months-old) *O. degus* display a larger individual variability in the fraction of wrong choices (%errors) in the radial arm maze (RAM) than young individuals (7–10 month-old). **(E)** In aged *O. degus* the mGluR-LTD does correlate with the behavioral score. The graph plots the mGluR-LTD averaged by individual against the individual’s behavioral score. Data points in **(A,B)** and boxes in **(C,D)** depict average ± SEM. Asterisks denote significance (*p* < 0.005). The number of mice and slices is indicated in parentheses). The blue lines in panels **(B,E)** are drawn for visual purposes; the *p* values in panels **(B,E)** correspond to the Pearson correlation. Calibration bars in panel **(A)**: 10 ms, 1 mV.

As mentioned, aging *O. degu* exhibit clear cellular and molecular features common to the early stages of AD. Therefore, we examined how aging affects mGluR-LTD in O. degu and used the same induction approach described above for the APP/PS1 Tg mice. We quantified mGluR-LTD of the CA3➔CA1 synapse in slices from individuals ranging from 7 to 94 months of age, which covers the full life expectancy of adult O. degu. As shown in Figure 3A, in slices from young individuals, aged 7–18 months, mGluR-LTD induction was robust (74.1 ± 3.8, *n* = 8 degu, 28 slices), whereas in slices from older individuals, aged 49–85 months, GluR-LTD induction was negligible (100.0 ± 3.4, *n* = 11 degu, 37 slices). These differences were significant (*p* < 0.001 Mann–Whitney test). Importantly, a more detailed analysis indicated that the average magnitude of mGluR-LTD correlated with the age of the animals (Figure 3B). In slices from young individuals, we confirmed that this form of LTD is blocked by the combination of antagonists of mGluR5 used in Figure 1D (Figure 3C; Control: 27.2 ± 5.9 change, *n* = 10 slices; MPEP-LY: 5.8 ± 3.5, *n* = 6 slices; *p* = 0.011; Mann–Whitney test). On the other hand, concerning the status of mGluR-LTP, the protocols developed for rat and mouse did induce NMDAR-independent LTP in *O. degu*, but with a very low success yield, inadequate to address developmental changes and to correlate them with behavior.

Finally, we determined whether the diminished capacity for mGluR-LTD in aged *O. degu* has a behavioral correlate. To that end, we tested *O. degu* individuals in a radial maze task, developed for rats (Chappell et al., 1998), where subjects get rewarded by avoiding exploring previously visited arms (see methods). Performance in this task was quantified as the percentage of retroactive memory (RAM) error, basically, the percentage of revisited arms. First, we confirmed that the% of RAM error is lower in young (7.7 ± 0.4 month) than in old (64.2 ± 4.2 month) individuals (Young: 37.4 ± 3.1%, *n* = 9; old: 57.2 ± 7.6, *n* = 11; *t*-test: *p* = 0.039; Figure 3D). Subsequently, we determined the average mGluR-LTD *ex vivo* in the aged individuals behaviorally characterized. As shown in Figure 3E, the behavioral score (% RAM error) and the average mGluR-LTD magnitude per individual exhibited a significant linear correlation.

## Discussion

Previously we and others showed that preserving learning in aged rats and mice requires the enhancement of mGluR-dependent plasticity in CA3➔CA1 synapses (Lee et al., 2005; Boric et al., 2008; Lu et al., 2019). Here we found that in APP/PS1 mice the induction of mGluR-LTD and mGluR-LTP, both robust in young 3-month-old individuals, virtually disappears by the 8-month of age. We also found a profound loss of mGluR-LTD in aging *O. degu* that correlates with diminished performance in a radial maze. These findings further support the notions that mGluR-dependent forms of plasticity are necessary for learning during aging and their early impairment in the path to AD. The results also underscored different age-related trajectories toward cognitive decline among rodent species: in aged Long-Evans rats impaired learning associates with failure to enhance mGluR-dependent plasticity (Lee et al., 2005; Boric et al., 2008), whereas in aged *O. degu* impaired learning correlates with loss of mGluR-LTD.

As mentioned, few studies have explored the status of mGluR-LTD in AD models. Intriguingly, these few studies document discrepant findings. The first reports indicated a diminished induction of mGluR-LTD in the CA3➔CA1 path in 12-15 month-old APP/PS1 mice when attempted with bath application of the type I agonist DHPG (Yang et al., 2016, 2021). This substantially agrees with the virtual absence of LFS-induced mGluR-LTD that we report in Figure 1. The difference in the magnitude of impairment, partial versus complete, might relate to the protocol used, as bath applied agonist would likely activate maximally the full complement of mGluR5 receptors, whereas ppLFS would activate only those accessible to endogenously released glutamate. Notably, however, a recent study reported that ppLFS-induced mGluR-LTD in CA3➔CA1 is enhanced, not reduced, in 7month APP/PS1 mice (Privitera et al., 2022). The enhancement of mGluR-LTD was found to be transient as it declined back to wild-type levels by the 13th month of age (Privitera et al., 2022). In sum, opposite outcomes were obtained in the same model of AD (APP/PS1), with the same induction paradigm (ppLFS) applied at comparable ages (7 and 8 months).

These seemingly discrepant outcomes are each concordant with distinct segments of the literature. In the case of the reduction in mGluR-LTD, which also associates with diminished learning performance in aged Degu (Figure 3), the finding dovetails well with the notion that an enhancement of mGluR-dependent plasticity is necessary to compensate for the loss of NMDAR-dependent plasticity during non-pathological aging (Menard and Quirion, 2012a,b). In addition, the normal expression of mGluR5 protein in the face of null mGluR-LTD/P at 8 months APP/PS1 (Figure 2), and the previous finding that wild-type levels of mGluR-LTD can be restored by targeting the kinase PERK (Yang et al., 2016), are both consistent with the notion that the uncoupling of type 1 mGluRs is a main contributing factor to age-related cognitive decline (Nicolle et al., 1999). However, in the 5XFAD mouse, a more extreme model of AD, studies report a progressive loss of functional mGluR5Rs (Lee et al., 2019). Nevertheless, whether via uncoupling or loss of the receptors, the deleterious consequences of a reduced mGluR5 functionality complement the observation that increasing mGluR5 function with allosteric modulators ameliorates some pathological signatures in other mouse models of AD (Bellozi et al., 2019). Finally, the loss of mGluR-LTD is consistent with the early observation that *in vitro* overexpression of APP occludes the induction of DHPG-LTD (Hsieh et al., 2006), although the occlusion scenario seems unlikely in the APP/PS1 mice because basal synaptic strength is normal in these mice (Megill et al., 2015). On the other hand, the case of an increased mGluR-LTD in the APP/PS1 mice (Privitera et al., 2022) agrees well with the observation of elevated mGluR-LTD in rats that model AD via intraventricular injection of synthetic Aβ peptide (Hu et al., 2022). Moreover, the idea that enhanced mGluR-LTD contributes to AD is in line with studies reporting that blocking mGluRs improve cognitive deficits in AD models (Um et al., 2013; Hamilton et al., 2016; Sanderson et al., 2016; Haas et al., 2017; Kazim et al., 2017). See (Brody and Strittmatter, 2018) for a review), and with the observation that Aβ oligomers enhance clustering and functionality of mGluR5’s (Renner et al., 2010). Conceptually, the idea of enhanced mGluR-LTD impairing cognition in AD resonates with the well-recognized negative effects of enhanced mGluR-LTD, and mGluR5 functionality, on cognition in a mouse model of Fragile X syndrome (Luscher and Huber, 2010; Ribeiro et al., 2017; Wilkerson et al., 2018). Indeed, the potential negative consequences of excessive mGluR-LTD were first noted in its initial characterization (Huber et al., 2000, 2002; Bear et al., 2004), when defining features became its dependence on protein synthesis and its excess in Fragile X mice.

A widely held tenet is that plasticity mechanisms need to be at a strict permissive range for optimal neural function. Hence excessive and diminished mGluR-LTD can both be deleterious for cognition. The conundrum is how these two opposite conditions might show up in the same mouse line, the APP/PS1. Differences in mice age at the time of testing seem unplausible. Like the magnitude of NMDAR-dependent plasticity (Lee et al., 2005; Boric et al., 2008; Megill et al., 2015) the magnitude of mGluR-LTD declines with age (in mice and degu), but in a monotonous trend. To account for the reported discrepancies between us and the Privitera et al. (2022) report the changes in the APP/PS1 need to fluctuate in a roller-coaster manner, from normal at 3 months of age, to excessive at 7 months, disappearing by 8 months, and finally recovering to wild type levels by the 13th month. Two methodological differences were noted between the two labs where the sucrose dissection buffer was used in our lab and the pharmacological blockade of synaptic inhibition was used by Privitera et al. (2022). It is unclear, however, how these treatments could affect preferentially, and transiently, mGluR-LTD only in the APP/PS1 mice but not in the wild types, which expressed comparable levels in both labs.

It is important to note in the APP/PS1 mice the discrepancies in synaptic plasticity are not restricted to mGluR-LTD and multiple studies also report disparate results on NMDAR-dependent LTP (Marchetti and Marie, 2011). These discrepancies in LTP have been attributed in part to abnormal developmental metaplasticity of LTP in APP/PPS1 (Megill et al., 2015). Metaplasticity refers to feedback mechanisms that dynamically maintain the induction and gain of synaptic plasticity within ranges that ensure stability and optimal processing of the neural network (Abraham and Bear, 1996). Importantly, altered metaplasticity is well-documented for NMDAR-dependent forms (Singh et al., 2019; Sanderson et al., 2021). It is conceivable that mGluR-dependent plasticity, like NMDAR-LTP, is also normally governed by that type of feedback mechanism. Disruption of metaplasticity might destabilize mGluR-LTD induction such that, which can drift to extremely high or low values. The particular endpoint of the drift could depend on external contingencies like colony conditions or husbandry protocols. We surmise that is worth considering that altered metaplasticity contributes to discrepancies in plasticity reported by different labs. In that vein, perhaps in AD, the fundamental alteration might be the control of plasticity, not plasticity itself.

## Data availability statement

The raw data supporting the conclusions of this article will be made available by the authors, without undue reservation.

## Ethics statement

Animal protocols reviewed and approved by Universidad de Valparaiso and Johns Hopkins University.

## Author contributions

GV, AA, AI, and CS performed the experiments. H-KL, MG, AP, and AK provided the funding. All authors contributed to design and writing.
